# Blind-noise image denoising with block-matching domain transformation filtering and improved guided filtering

**DOI:** 10.1038/s41598-022-20578-w

**Published:** 2022-09-28

**Authors:** Hongbin Jia, Qingbo Yin, Mingyu Lu

**Affiliations:** Intelligent Technology Research Center, College of Information Sciences and Technology, Dalian Maritime University, No.1 LingHai Road, Dalian, 116026 Liaoning Province China

**Keywords:** Computational science, Computer science

## Abstract

The adaptive block size processing method in different image areas makes block-matching and 3D-filtering (BM3D) have a very good image denoising effect. Based on these observation, in this paper, we improve BM3D in three aspects: adaptive noise variance estimation, domain transformation filtering and nonlinear filtering. First, we improve the noise-variance estimation method of principle component analysis using multilayer wavelet decomposition. Second, we propose compressive sensing based Gaussian sequence Hartley domain transform filtering to reduce noise. Finally, we perform edge-preserving smoothing on the preprocessed image using the guided filtering based on total variation. Experimental results show that the proposed denoising method can be competitive with many representative denoising methods on the evaluation criteria of PSNR. However, it is worth further research on the visual quality of denoised images.

## Introduction

Among many methods of image denoising, domain transformation filtering is one of the most important research projects^1^. The idea of domain transform filtering is to transform the noise image from the spatial domain to the transform domain. Then, the transform coefficients are processed by the inherent characteristics of the transform domain to reduce noise. Finally, the output image is reconstructed by inverse domain transform^2,3^. Many excellent denoising methods based on domain transformation filtering have been proposed for decades, such as Wavelet Transform^4^, Multiscale Geometric Analysis (MGA)^5–8^, Block-matching and 3D-collaborative filtering algorithm (BM3D)^9^, The Principal Component Analysis with Local Pixel Grouping (LPG-PCA)^10^, etc. And there are methods for domain transformation using dictionary learning^11–13^, etc. These methods achieve excellent denoising performance.

Among the numerous methods, BM3D effectively combines the non-local similarity of images and domain transform filtering, and achieves good denoising performance. Through these two key operations, similar blocks in an image are grouped and aggregated into 3D groups. Then, the grouped blocks are realized domain transformation (sparse representation), and get individual estimates through collaborative filtering. Finally, the denoised image is obtained by aggregation. The excellent structure and denoising effect make BM3D one of the best denoising methods and the evaluation standard of denoising effect, it is one of the methods that must be learned in the research of image denoising. Moreover, BM3D is well worth further research and improvement. For example, Zhong et al. proposed to modified BM3D algorithm by nonlocal centralization prior^14^, Feng et al. improved BM3D algorithm in terms of Gaussian threshold and angular distance respectively^15^, In addition, various researches have been proposed to improve the denoising effect, retain more image details, enrich the application mode of BM3D, and so on^16–19^.

In this paper, in order to make BM3D have better practicability and denoising effect, we improve BM3D in three aspects: adaptive noise variance estimation, domain transformation filtering and nonlinear filtering. Unlike images with known noise variance in experiments, the noise variance of real noisy images is unknown, i.e. blind noise images. First, the noise variance value is a precondition for the application of BM3D, which plays a very important role in collaborative filtering and weight calculation. Second, the wavelet transform cannot fully utilize the geometric features of the image, so it cannot represent the image sparsely effectively, which affects the denoising effect of the domain transformation filtering. Third, the Wiener filter for denoising in the second stage of BM3D is a linear filter, which will cause the destruction of image edges in the filtering process.

In the study of noise variance estimation, the error of almost all estimation methods increases with the increase of noise variance. In order to get more accurate estimation, we improve Principal Component Analysis (PCA) noise variance estimation method with multilayer wavelet decomposition. It is found that after multi-layer wavelet decomposition, there are multiple approximate representations of image in these low-frequency wavelet subbands, which filter the information of image contour and texture. Moreover, the feature distributions of image and noise in the wavelet subband are different, and the noise in the wavelet subband is consistent with the feature distribution in the image. Based on these observation, we try to estimate the noise variance more accurately. We first perform multi-layer wavelet decomposition on the image, then select appropriate layers and estimate the noise variance of each layer, and finally synthesize the results of each layer to obtain the noise variance estimate.

For better sparse representation of images, inspired by Compressed Sensing (CS)^20^, we propose a domain transformation filtering method. According to the construction of the sensing matrix in CS, we combine the radial basis function (RBF) kernel of the Gaussian process with the discrete Hartley transform (DHT) to construct the Gaussian sequence Hartley transform (GHT), which is used to realize the domain transformation^21^. Then, the modified threshold shrinkage based on basis pursuit denoising (BPDN) is used to filter the noise coefficients^22^, finally the preliminary denoised image is obtained by inverse transformation and restoration of filtered coefficients, which can be defined as the basic estimation.

In order to preserve the image edges as much as possible while denoising, we use guided filter (GF) instead of Wiener filter to remove residual noise in the preliminary denoised image^23^. Guided filter is an excellent edge-preserving filter, but the quality of the guided image will seriously affect the filtering performance. In order to get good denoising effect, we improve the guided filter by optimizing the relationship between the guided image and the input image with the total variation (TV) regularization term^24^.

The adaptive noise variance estimation makes the proposed method more practical and contributes to the blind noise image denoising. And then, block-matching domain transform filtering and improved guided filtering are successively used for image denoising. Experimental results show that the proposed method has better visual effect and higher PSNR compared with BM3D. Compared with other benchmark and representative image denoising methods, the proposed method also has strong competitiveness.

The remainder of this paper is organized as follows. In Sect. “Related works”, we briefly introduce the related works. In Sect. “The proposed method”, we present the proposed method. Experimental results are shown in Sect. “Experimental results” and conclusion is given in Sect. “Conclusion”.

## Related works

### Noise variance estimation

In image denoising, noise variance estimation is one of the most fundamental problems and an important factor in most denoising methods. To obtain accurate estimation, many excellent noise variance estimation methods have been proposed over the years, among which PCA-based noise variance estimation is a very outstanding method^25^. In the PCA-based noise variance estimation, it is considered that the image mean eigenvalues converge to the noise variance:

1 $${\rm E}\left( {\left| {{\overline{\lambda }} - \sigma^{2} } \right|} \right) = O\left( {{\raise0.7ex\hbox{${\sigma^{2} }$} \!\mathord{\left/ {\vphantom {{\sigma^{2} } {\sqrt N }}}\right.\kern-\nulldelimiterspace} \!\lower0.7ex\hbox{${\sqrt N }$}}} \right),{ }N \to \infty$$

where $\overline{\uplambda }$ is the average value of image eigenvalues, ${\sigma }^{2}$ is the noise variance value of image, big $O$ notation means that $\exists C$, $\exists {N}_{0}$ such that $\forall N\ge {N}_{0}\mathrm{\rm E}\left(\left|\overline{\uplambda }-{\sigma }^{2}\right|\right)\le C{\sigma }^{2}/\sqrt{N}$, and $C$ does not depend on the distribution of image and noise.

**Figure 1:**
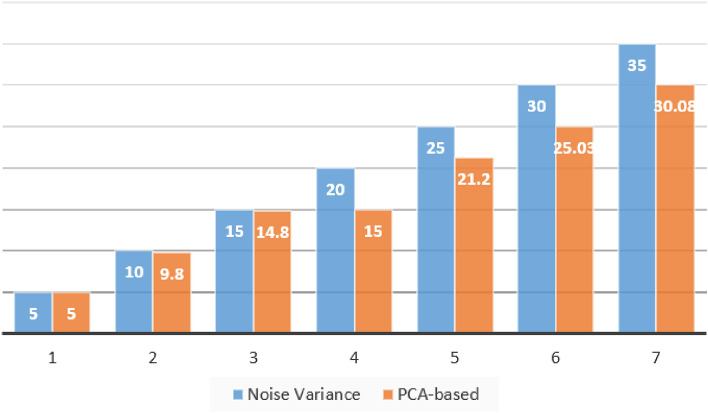
The PCA-based noise variance estimation. Added noise variance on the left, PCA-based estimates on the right.

However, with the increase of noise variance, the error of noise variance estimation increases with the increase of noise variance, as shown in Fig. 1. This is because the distribution difference between noise and image feature has become smaller, especially the high-frequency information in the image, and it is difficult to distinguish noise from image texture.

### BM3D and wavelet transform filtering

**Figure 2:**
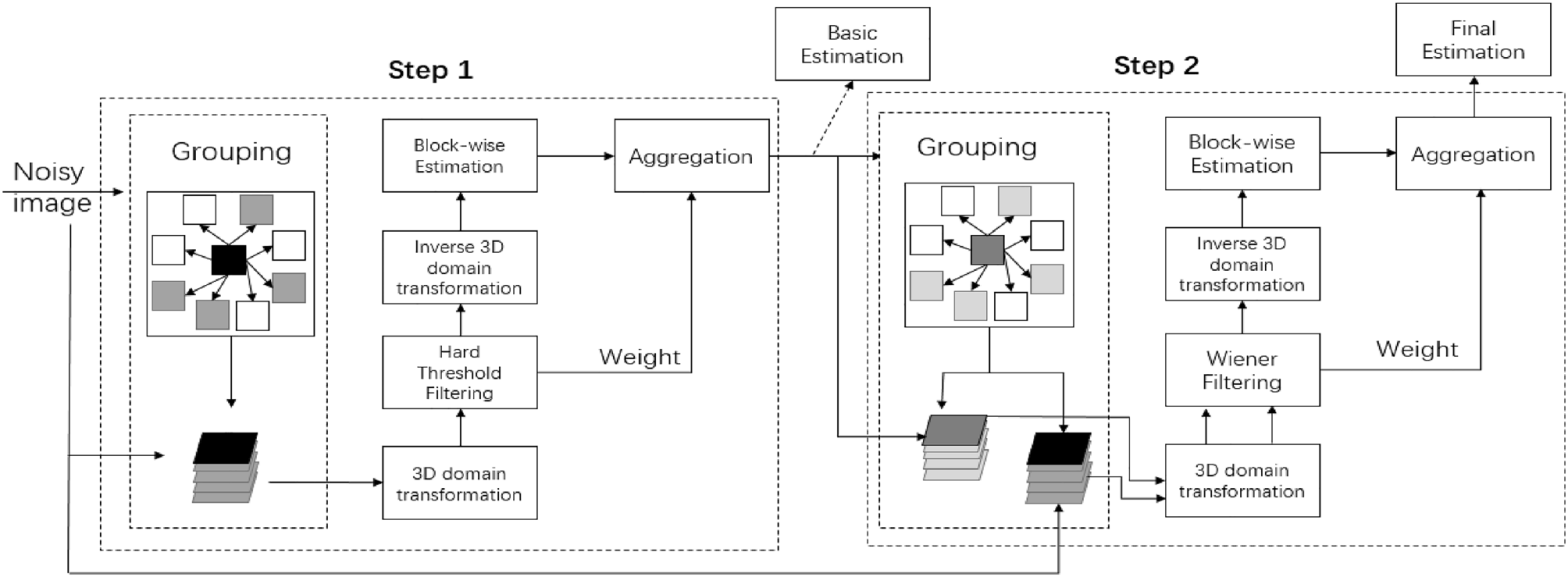
BM3D flow chart.

BM3D reduces noise through two stages: Basic estimation and Final estimation. Grouping, collaborative filtering, and aggregation are performed sequentially at each stage. Similar blocks are grouped and stacked into 3D groups in Grouping. These 3D groups are then transformed from spatial domain to wavelet domain by wavelet transform, and the third-dimension transform is performed by Hadamard-transform. After that, threshold shrinkage filtering and Wiener filtering are performed in two stages to filter the noise coefficients, which is called collaborative filtering. After all blocks have been processed, the estimates of all the overlapping blocks were weighted average and aggregated to obtain an exact estimate of image. The flow chart is shown in Fig. 2.

An important reason for the great success of signal processing based on wavelet analysis in many scientific research fields is that it can sparsely represent bounded signals or variation functions. After wavelet transform, the wavelet coefficients corresponding to the signal contain important information, and the amplitude is large and the number is small. The wavelet coefficients corresponding to the noise are uniformly distributed, with small amplitude and large number^4^. Since the coefficients corresponding to the signal can be sparsely represented with fewer coefficients, domain transformation can generally be considered as a sparse representation. Then, with the threshold shrinkage filtering, it is possible to preserve image information as much as possible while denoising. However, the wavelet transform extended from one-dimensional discrete wavelet transform to two-dimensional image processing has limited directionality, cannot fully utilize the geometric features of images, and cannot perform sparse representation particularly effectively. As a result, the feature distribution of noise and image cannot be distinguished effectively in the traditional wavelet transform domain, and the denoising effect achieved by threshold shrinkage is not very good.

### Wiener filtering and guided filtering

After domain transformation filtering, there is still noise with large coefficient in the image. In order to filter the remaining noise, wiener filtering is used in the second stage of BM3D. If the image model with noise is assumed to be $y\left(u,v\right)=H\left(u,v\right)*x\left(u,v\right)+n(u,v)$, Wiener filtering can be expressed as follows:

2 $$\hat{y}\left( {u,v} \right) = {\raise0.7ex\hbox{$1$} \!\mathord{\left/ {\vphantom {1 {H\left( {u,v} \right)}}}\right.\kern-\nulldelimiterspace} \!\lower0.7ex\hbox{${H\left( {u,v} \right)}$}}\left( {\frac{{\left| {H\left( {u,v} \right)} \right|^{2} }}{{\left| {H\left( {u,v} \right)} \right|^{2} + {\raise0.7ex\hbox{${S_{\sigma } \left( {u,v} \right)}$} \!\mathord{\left/ {\vphantom {{S_{\sigma } \left( {u,v} \right)} {S_{x} \left( {u,v} \right)}}}\right.\kern-\nulldelimiterspace} \!\lower0.7ex\hbox{${S_{x} \left( {u,v} \right)}$}}}}} \right)y\left( {u,v} \right)$$

where $x\left(u,v\right)$ is the clean image, $H\left(u,v\right)$ is the degenerative process, $y(u,v)$ is the noise image, $*$ is the convolution process, $n(u,v)$ is the additive noise.${\left|H\left(u,v\right)\right|}^{2}={H}^{*}\left(u,v\right)H\left(u,v\right)$ and ${(\cdot )}^{*}$ is the complex conjugate, ${S}_{\sigma }\left(u,v\right)={|n\left(u,v\right)|}^{2}$ and ${S}_{x}\left(u,v\right)={|x\left(u,v\right)|}^{2}$, ${S}_{\sigma }\left(u,v\right)/{S}_{x}\left(u,v\right)$ is the Noise signal power ratio, $\widehat{y}(u,v)$ is the image after wiener filtering. Unfortunately, as a linear spatial filtering method, wiener filtering not only removes noise, but also corrupts image information.

The idea of guided filtering is to use a guided image to generate weight, so as to process the input image. This process can be expressed as

3 $$q_{i} = \sum\limits_{j} {W_{{i,j}} } \left( I \right)p_{j}$$

where *q* is the output image, *I* is the guided image and *p* is the input image. $i$ and $j$ are the index of pixels in the image. An important assumption of guided filtering is that there is a local linear relationship between the output image and the guided image in a local window ${w}_{k}$, the linear relationship can be expressed as

4 $$q_{i} = a_{k} I_{i} + b_{k} ,\forall i \in w_{k}$$

${a}_{k}$ and ${b}_{k}$ are linear coefficients in ${w}_{k}$, their values are constant. The required coefficient ${a}_{k}$ and ${b}_{k}$ should minimize the difference between *p* and *q*, then a cost function is defined as

5 $$E\left( {a_{k} ,b_{k} } \right) = \mathop \sum \limits_{{i \in W_{k} }} \left[ {\left( {a_{k} I_{i} + b_{k} - p_{i} } \right)^{2} + \epsilon a_{k}^{2} } \right]$$

where $\upepsilon$ is the regularization parameter that prevents ${a}_{k}$ from becoming too large. Accordingly, the coefficients ${a}_{k}$ and ${b}_{k}$ are computed as

6 $$a_{k} = \frac{{\frac{1}{\left| w \right|}\mathop \sum \nolimits_{{i \in W_{k} }} I_{i} p_{i} - \mu_{k} \overline{{p_{k} }} }}{{\sigma_{k}^{2} + \epsilon }}$$

7 $$b_{k} = \overline{{p_{k} }} - a_{k} \mu_{k}$$

where ${\mu }_{k}$ and ${\sigma }_{k}^{2}$ are the mean and variance of the guided image in ${w}_{k}$, $|w|$ is the total number of pixels, $\overline{{p }_{k}}$ is the mean pixel value of the input image. Since the window has dimensions, a pixel will be calculated with linear coefficients in different windows, and different windows have different output values. Therefore, the values of ${a}_{k}$ and ${b}_{k}$ need to be averaged

8 $$q_{i} = \overline{{a_{k} }} I_{i} + \overline{{b_{k} }} ,\forall i \in w_{k}$$

As can be seen, the pixel value of the output image mainly depends on the guided image, it is considered that the quality of guided image determines the filtering effect. If the noise image is used as the guided image, the noise in guided image will cause the wrong weight and inaccurate gradient, and some noise coefficients will be amplified.

## The proposed method

### Adaptive noise variance estimation

According to Donoho’s theory^4^, when the noise image is subjected to wavelet decomposition, the wavelet coefficients corresponding to noise are evenly distributed at each scale, and the amplitude of the coefficient decreases with the increase of the scale. Moreover, the characteristic distribution of noise in wavelet subband and image is consistent. Based on the above, we assume that the noise variance can be estimated more accurately by the approximate representation in the first n-layers of low-frequency subbands. To test this hypothesis, we conducted a large number of simulation experiments. We calculate the noise variance of the approximate representation at different scales for noise images with known variance. An example is presented in Table 1 (the image ‘Boat’ is taken and the noise variance is set to 20).

**Table 1:** Noise variance in multilayer wavelet subbands (${\sigma }^{2}$ = 20).

Image	Subband	1-level	2-level	3-level	4-level	5-level
Boat	LL	19.43	20.10	20.70	32.20	78.14
	HL	18.88	18.72	20.73	31.01	92.48
	LH	18.72	18.99	22.12	38.79	82.37
	HH	19.68	17.24	20.08	14.41	22.24

The improved estimation method is as follows: the noise image is decomposed through multi-layer wavelet, then the noise variance of each layer is estimated by PCA-based estimation method, and finally the data of multi-layer is synthesized to get the final estimation^25^. However, it can be seen from Table 1 that only the first few layers of data are effective, and the effective layer of different images is different. So it is necessary to choose the appropriate level when applying.

**Figure 3:**
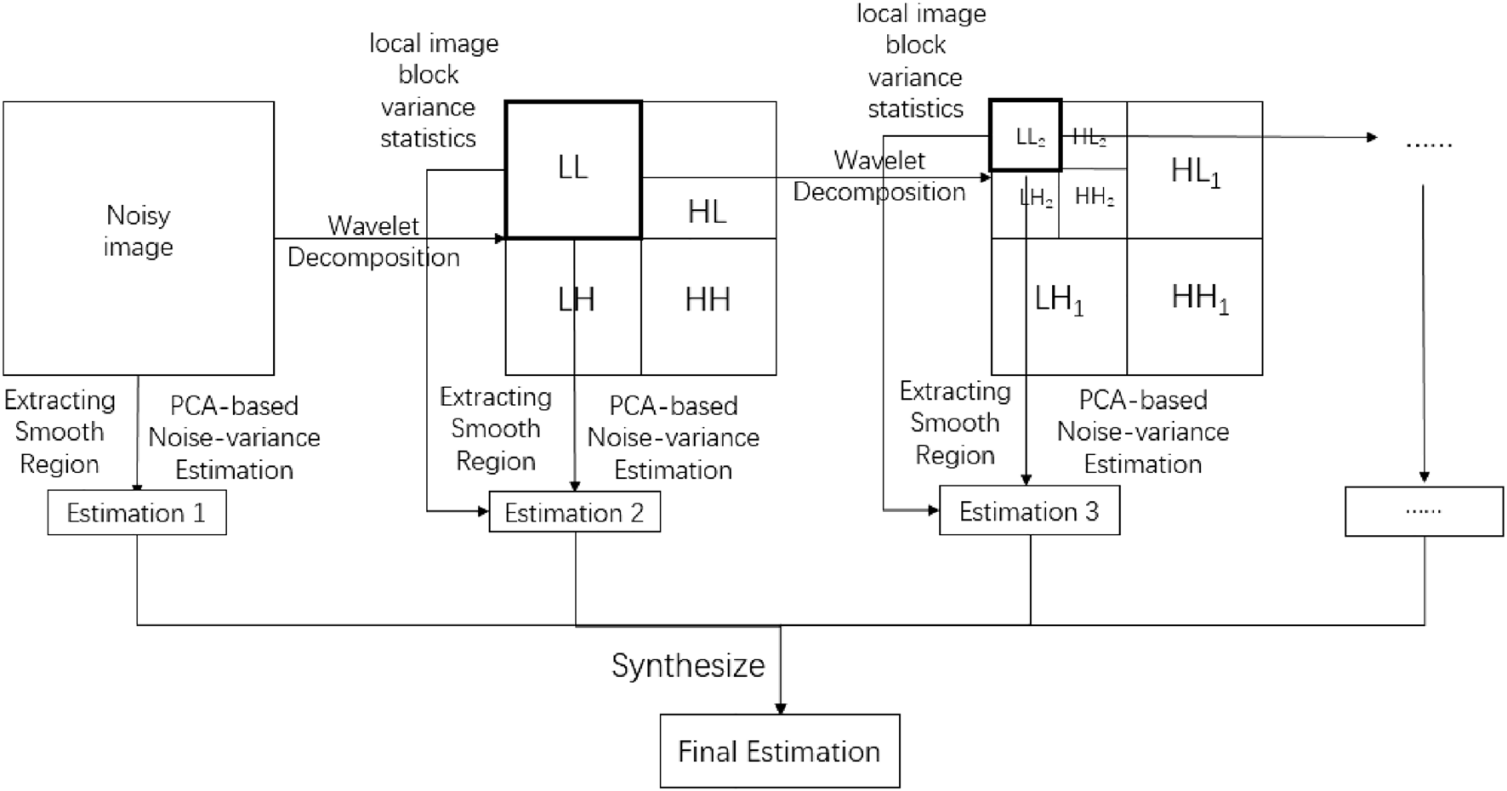
Flow chart of the improved PCA-based noise variance estimation method.

As described in Donoho-robust noise variance estimation method^5^, the noise coefficients are concentrated in the high frequency subband after wavelet decomposition, and the noise variance estimation is defined as

9 $${\upsigma } = {\text{median}}\left( {W_{HH} } \right)/0.6745$$

where the value 0.6745 is a highly robust attenuation value of noise coefficient amplitude. In the noise variance estimate of the proposed method, we take the robust attenuation value into account. When the coefficient scaling ratio of adjacent layer exceeds this value, the data in this layer is considered to be distorted, and the previous layer is selected for the constraint layer. In order to avoid the layer selection error caused by the PCA-based estimation error alone, we also use the method of image local block variance statistics to calculate the coefficient amplitudes under different wavelet decomposition scales and select the appropriate layer. To more easily understand the improved noise variance estimate method, the flow chart of estimate method is shown in Fig. 3. And there are some problems needing attention in the improved PCA-based noise variance estimation method:

1. In some wavelet subband, the coefficient amplitude varies greatly between different layers, it is difficult to obtain accurate estimation. In order to select the appropriate wavelet basis, we compare the orthogonality, compact support, support width, vanishing moment and other characteristics of various wavelet basis, and select the wavelet basis ‘Symlets’.
2. The texture, contour and other details in the image will greatly reduce the estimation. In the smooth region, the influence of image details can be reduced so that the noise variance can be better calculated^26, 27^.

### Block-matching domain transformation filtering

In this section, we introduce a block-matching domain transformation filtering based on compressed sensing (CS). Compressed sensing is a technique to search for sparse solutions of underdetermined linear systems, it is used to obtain and reconstruct sparse or compressible signals. Compared with Nyquist’s sampling theory, compressed sensing can recover the whole original signal from fewer measured values through the sparse characteristic of the signal. It should be noted that compressed sensing does not break Nyquist's limit. In compressed sensing, sampling and compression of signal are carried out simultaneously, rather than sampling and then compression in Nyquist's theorem.

In compressed sensing, it is considered that if the signal can be sparsely represented by the transformation matrix of the domain transformation, it means that the signal is sparse in this transform domain, and it can be transformed from a high-dimensional space to a low-dimensional space by a measurement matrix independent of the transformation matrix. Then, by solving an optimization problem, the original signal can be reconstructed from these projections with a high probability, which can be expressed as

10 $$y = {\Phi }x$$

where ${\Phi }$ is the measurement matrix, $x$ is the signal. However, $x$ is usually not sparse and needs to be represented sparsely with a sparse basis

11 $$x = \Psi \theta$$

${\Psi }$ is the transformation matrix, then the function (10) can be written as

12 $$y = \Phi \Psi \theta$$

where the combination of $\Phi$ and $\Psi$ can form the sensor matrix in compressed sensing.

It can be concluded from the above that there are two conditions for compressed sensing, which are also the conditions for constructing domain transformation:

1. Sparsity. When the signal is sparse or approximately sparse, it can perform compressed sensing and restore the signal with fewer measured value.
2. Incoherence. To ensure convergence, the measurement matrix should satisfy Restricted Isometry Property (RIP)^28^, that is, for any strictly k-sparse vector C, the following functional constraints should be satisfied.
  13 $$1 - \varepsilon \le \frac{{\left| {\left| {{\Phi c}} \right|} \right|_{2} }}{{\left| {\left| {\text{c}} \right|} \right|_{2} }} \le 1 + \varepsilon ,\varepsilon > 0$$

Baraniuk proved that the equivalent condition of RIP is that the measurement matrix and the sparse matrix should be independent^29^. And Candès and Tao proved that the independent and identically distributed Gaussian random measurement matrix can be a universal measurement matrix.

In the sparse representation of signal, an appropriate sparse basis can minimize the number of signal sparseness, which is conducive to improve the signal acquisition rate and reducing the resources occupied by storage and transmission. We choose the Discrete Hartley transform (DHT) for sparse representation in the proposed domain transformation. The reason is that DHT has no complex number operation and the calculation is small. The forward transformation and the inverse transformation are the same, which are the integral transformation of the strict reciprocal of a pair of real numbers. The advantages of DHT make it very suitable for spectral analysis and convolution operation of real data, and also contribute to the research of domain transformation about sensor matrix. The function of DHT is defined as

14 $$X_{H} \left( k \right) = \sqrt {{\raise0.7ex\hbox{$1$} \!\mathord{\left/ {\vphantom {1 N}}\right.\kern-\nulldelimiterspace} \!\lower0.7ex\hbox{$N$}}} \mathop \sum \limits_{n = 0}^{N - 1} x\left( n \right)cas\left( {\frac{2\pi }{N}nk} \right)$$

where $$cas\left( {\frac{2\pi }{N}nk} \right) = \cos \left( {\frac{2\pi }{N}nk} \right) + \sin \left( {\frac{2\pi }{N}nk} \right)$$, $$k = 0,1,2 \ldots ,N - 1$$.

According to the sensor matrix, the proposed domain transformation is constructed using Gaussian process combined with sparse matrix. Gaussian process is determined by its mathematical expectation and covariance function, and its properties are closely related to its covariance function, while some covariance functions in Gaussian process are kernel functions. The mathematical expectation of a stationary Gaussian process is a constant, so the Gaussian process is completely defined by the kernel function. Therefore, the proposed domain transformation can be simplified as finding a suitable kernel function and combining the sparse function to construct the domain transformation function. And we choose Radial Basis Function (RBF) kernel for the research.

15 $$\kappa \left( r \right) = {\text{exp}}\left( { - \frac{{r^{2} }}{{2l^{2} }}} \right)$$

where $r$ is the difference between the two eigenvectors, $l$ is the width parameter of the function.

Accordingly, the positive transformation function of the proposed domain transformation is obtained as

16 $$X_{GHT} \left( k \right) = \sqrt {{\raise0.7ex\hbox{$1$} \!\mathord{\left/ {\vphantom {1 N}}\right.\kern-\nulldelimiterspace} \!\lower0.7ex\hbox{$N$}}} \mathop \sum \limits_{n = 0}^{N - 1} x\left( n \right)\exp \left[ { - \frac{{\left( {cas\left( {\frac{2\pi }{N}nk} \right)} \right)^{2} }}{2}} \right]$$

where $cas\left( {\frac{2\pi }{N}nk} \right) = \cos \left( {\frac{2\pi }{N}nk} \right) + \sin \left( {\frac{2\pi }{N}nk} \right),k = 0,1,2 \ldots ,N - 1$. The inverse transformation can be derived as

17 $$x\left( n \right) = \mathop \sum \limits_{n = 0}^{N - 1} X_{GHT} \left( k \right)\exp \left[ {\frac{{\left( {cas\left( {\frac{2\pi }{N}nk} \right)} \right)^{2} }}{2}} \right]$$

and $$cas\left( {\frac{2\pi }{N}nk} \right) = \cos \left( {\frac{2\pi }{N}nk} \right) + \sin \left( {\frac{2\pi }{N}nk} \right),n = 0,1,2 \ldots ,N - 1$$.

The pair of forward and inverse transformation functions constitute the domain transformation of Gaussian sequence Hartley transformation (GHT). In different transform domains, the feature distributions of noise and signal are different. In GHT, we need a corresponding threshold shrinkage method to filter the noise coefficients. In compressed sensing, in order to reduce the influence of noise in signal, the basis pursuit denoising (BPDN) model is proposed^22^. If the noise model is assumed to be

18 $$y = x + \sigma z$$

where $x$ is the clean image, $y$ is the noise image, $\sigma z$ is the noise affected by noise variance $\sigma$, the model of BPDN is

19 $$\mathop {\min }\limits_{a} \frac{1}{2}\left\| {y - {\Phi }} \right\|a_{2}^{2} + {\uplambda }\left\| a \right\|$$

The solution $a^{\left( \lambda \right)}$ is a function of the parameter ${\uplambda }$. It yields a decomposition into signal-plus-residual.

20 $$y = x^{\left( \lambda \right)} + r^{\left( \lambda \right)} m,\;x^{\left( \lambda \right)} = {\Phi }a^{\left( \lambda \right)}$$

where $\lambda$ controls the size of the residual. The variable $a$ can be split into its positive and negative parts, $a=u-v, u\ge 0,v\ge 0.$ These relationships are satisfied by ${u}_{i}={({a}_{i})}_{+}$ and ${v}_{i}={({-a}_{i})}_{+}$ for all i = 1,2…n. where ${(\cdot )}_{+}$ denotes the positive-part operator as ${(a)}_{+}=\mathrm{max}\{0,a\}$. Then there are ${||a||}_{1}={1}_{n}^{T}u+{1}_{n}^{T}v,$ where $1_{n} = \left[ {1,1 \ldots ,1} \right]^{T}$ is the vector consisting of $n$ ones. The function (19) can be rewritten as the following bound-constrained quadratic program (BCQP)

21 $$\mathop {\min }\limits_{u,v} \frac{1}{2}\left\| {y - {\Phi }\left( {u - v} \right)} \right\|_{2}^{2} + {\uplambda }(1_{n}^{T} u + 1_{n}^{T} v),{\text{ s}}.{\text{t}}.{ }u \ge 0,v \ge 0.$$

It can be written in more standard BCQP form

22 $$\mathop {\min }\limits_{z} c^{T} z + \frac{1}{2}z^{T} {\text{B}}z{\text{ subject to }}z \ge 0$$

where $$z = \left[ {u,v} \right]^{T} ,{\text{b}} = A^{T} y, c = \tau 1_{2n} + \left[ { - b,b} \right]^{T}$$, and $$B = \left[ {\begin{array}{*{20}c} {A^{T} A} & { - A^{T} A} \\ { - A^{T} A} & {A^{T} A} \\ \end{array} } \right]$$.

According to the BCQP form, then BPDN can be transformed into a perturbed linear programming problem

23 $$\mathop {\min }\limits_{{}} c^{T} x + \frac{1}{2}\left| {\left| p \right|} \right|^{2} {\text{ subject to Ax}} + {\sigma p} = {\text{b}},{\text{ x}} \ge 0$$

where A = ($\Phi ,-\Phi$), b = y, c = 1. Perturbed linear programming is a quadratic programming problem, but it retains a similar structure to linear programming. Then the denoising method with BPDN refers to minimizing the least square fit error plus a penalizing term^22^:

24 $$\mathop {\min }\limits_{a} \frac{1}{2}\left\| {x - {\Phi }a} \right\|_{2}^{2} + {\uplambda }\left\| a \right\|_{2}^{2}$$

the penalizing parameter $\lambda$ in BPDN optimization model can be set to the value

25 $$\lambda_{p} = {\upsigma }\sqrt {2{\text{log}}\left( p \right)}$$

where $p$ is the cardinality of the dictionary, and assuming the dictionary is normalized,. The threshold shrinkage function can be derived as

26 $$S\left( a \right) = \left\{ \begin{gathered} a\;\;a > |\lambda_{p} | \hfill \\ 0\;\;else \hfill \\ \end{gathered} \right.$$

**Figure 4:**
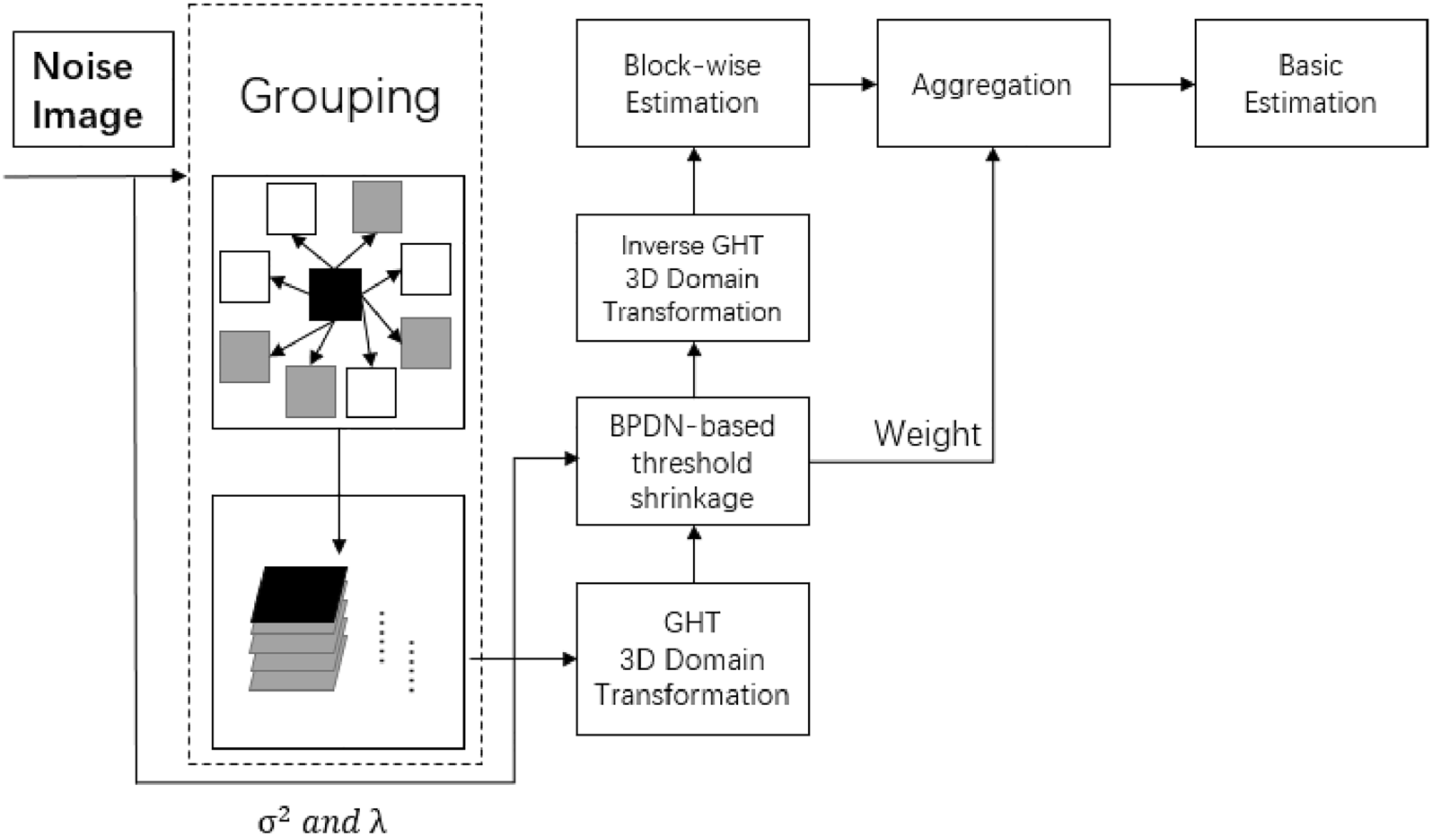
Flow chart of the proposed block-matching domain transformation filtering.

Setting threshold shrinkage as a hard-threshold can better retain image details in image denoising. The proposed domain transformation filtering replaces wavelet transform filtering, and the flow chart of the proposed block-matching domain transformation filtering is shown in Fig. 4.

### Improved guided filtering

As mentioned above, the deficiency of wiener filtering results in the decrease of image denoising effect, which is verified by the denoising results, as shown in Table 2. We can see from Table 2 that the PSNR of the final estimation is generally lower than the basic estimation. This is almost consistent with the analysis of the shortcomings about linear filtering, which causes image information damage while denoising. And the wiener filtering function in BM3D can be expressed as

27 $$f_{w} \left( \theta \right) = \theta \frac{{\left| {\hat{\theta }} \right|^{2} }}{{\left| {\hat{\theta }} \right|^{2} + \sigma^{2} }}$$

where $\theta$ is the noise image coefficient, $\widehat{\theta }$ is the coefficient of basic estimation, ${\sigma }^{2}$ is the noise variance. After Grouping and domain transformation filtering, wiener filtering is used to filter the array of noise image coefficient. But wiener filtering reduces both the noise coefficient and the image coefficient during Collaborative Filtering, resulting in the degradation of the image quality.

**Table 2:** The denoising results at each stage of BM3D.

Image	Noise variance	Noise image (PSNR)	Basic estimation (PSNR)	Final estimation (PSNR)
Boat	5	23.10	23.84	23.59
	15	18.43	21.99	20.69
	25	16.34	22.70	21.83
Cameraman	5	23.32	24.21	23.9
	15	18.65	22.51	21.2
	25	16.62	23.32	22.58

**Figure 5:**
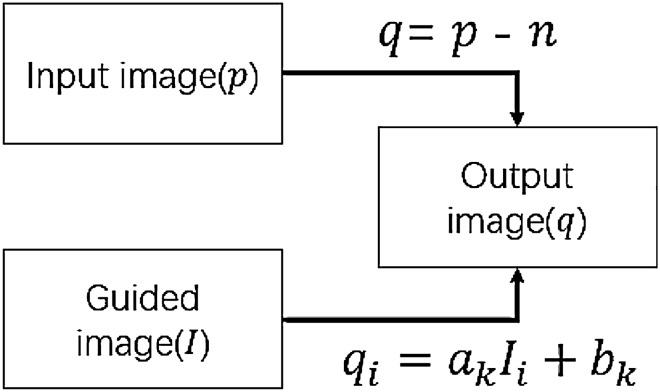
Guided filtering structure diagram.

To remedy this deficiency, we try to use the guided filtering to filter the remaining noise. Guided filtering is a kind of edge preserving filter that preserves image information as much as possible while denoising. Compared with other edge-preserving filter, guided filtering overcomes the problem of gradient flipping and has high computational efficiency. The structure diagram of guided filtering is shown in Fig. 5. However, there is only the noise image can be used as the guided image in image denoising, which seriously affect the performance of guided filtering. So we propose an improved guided filtering to get better denoising effect.

When we take the derivative of the function (4)

28 $$\nabla q = a_{k} \nabla I$$

This shows that when the gradient of the guide image changes locally, the gradient of the output image also changes at the corresponding position. And ${a}_{k}$ is the decisive factor in gradient calculation of guided filtering. In noise images, noise creates many extra gradients. In the image denoising method based on gradient prior, it is believed that the gradient (total variation) of an image is limited, and the image denoising problem can be transformed into a total variation optimization problem^30^. The mathematical definition of total variation (TV) is

29 $$V_{TV} \left( p \right) = \mathop \sum \limits_{i,j} \left| {p_{i + 1,j} - y_{i,j} } \right| + \left| {\left( {p_{i,j + 1} - p_{i,j} } \right)} \right|$$

where $p$ is the input image, $i$ and $j$ are the index of pixels. On the other hand, the function (6) of ${a}_{k}$ can be written as

30 $$a_{k} = \frac{1}{{\left| w \right|\cdot\left( {\sigma_{k}^{2} + \epsilon } \right)}}\mathop \sum \limits_{{i \in W_{k} }} I_{i} p_{i} - \mu_{k} \overline{{p_{k} }}$$

where ${\mu }_{k}$ and $\overline{{p }_{k}}$ are the mean of the guided image and the input image respectively, which can be regarded as ${I(\mu }_{k})$ and $p(\overline{{p }_{k}})$, that is, the related terms of the guided image and the input image. $\left|w\right|\cdot ({\sigma }_{k}^{2}+\upepsilon )$ is the scaling factor and its value is constant, therefore, ${a}_{k}$ can be regarded as an optimization model of the relationship between the input image $p$ and the guided image $I$. Then, the optimization model can be regarded as

31 $$R\left( {I,p} \right) = \mathop \sum \limits_{{i \in W_{k} }} I_{i} p_{i} - I(\mu_{k} )p(\overline{{p_{k} }} )$$

Based on the above, we believe that noise adversely affects the relationship model in (31), resulting in an abnormal increase in gradients and reducing the output image quality. Therefore, we proposed an improved guided filtering by using total variation to optimize the relationship of $a_{k}$

32 $$R_{TV} \left( {I,p} \right) = \left[ {\mathop \sum \limits_{{i \in W_{k} }} I_{i} p_{i} - I(\mu_{k} )p(\overline{{p_{k} }} )} \right] + \lambda_{sf} V_{TV} \left( I \right)$$

where ${\lambda }_{sf}$ is the smoothing factor of TV regularization term. Since guided filtering is a windowed operation, ${\lambda }_{sf}$ needs to be normalized, ${\lambda }_{sf}={\lambda }_{TV}/(size(image))$, and ${\lambda }_{TV}$ is a negative value to reduce the impact of noise in the guided image. In the improved guided filtering, the solutions of linear coefficients ${a}_{k}$ and ${b}_{k}$ are

33 $$a_{k} = \frac{{\frac{1}{\left| w \right|}\mathop \sum \nolimits_{{i \in W_{k} }} I_{i} p_{i} - \mu_{k} \overline{{p_{k} }} }}{{\sigma_{k}^{2} + \epsilon }} + \lambda_{sf} V_{TV} \left( I \right)$$

34 $$b_{k} = \overline{{p_{k} }} - a_{k} \mu_{k}$$

where $${\lambda }_{sf}={\lambda }_{TV}/(size(image))$$, $${\lambda }_{TV}<0$$.

### Adaptive blind-noise image denoising

**Figure 6:**
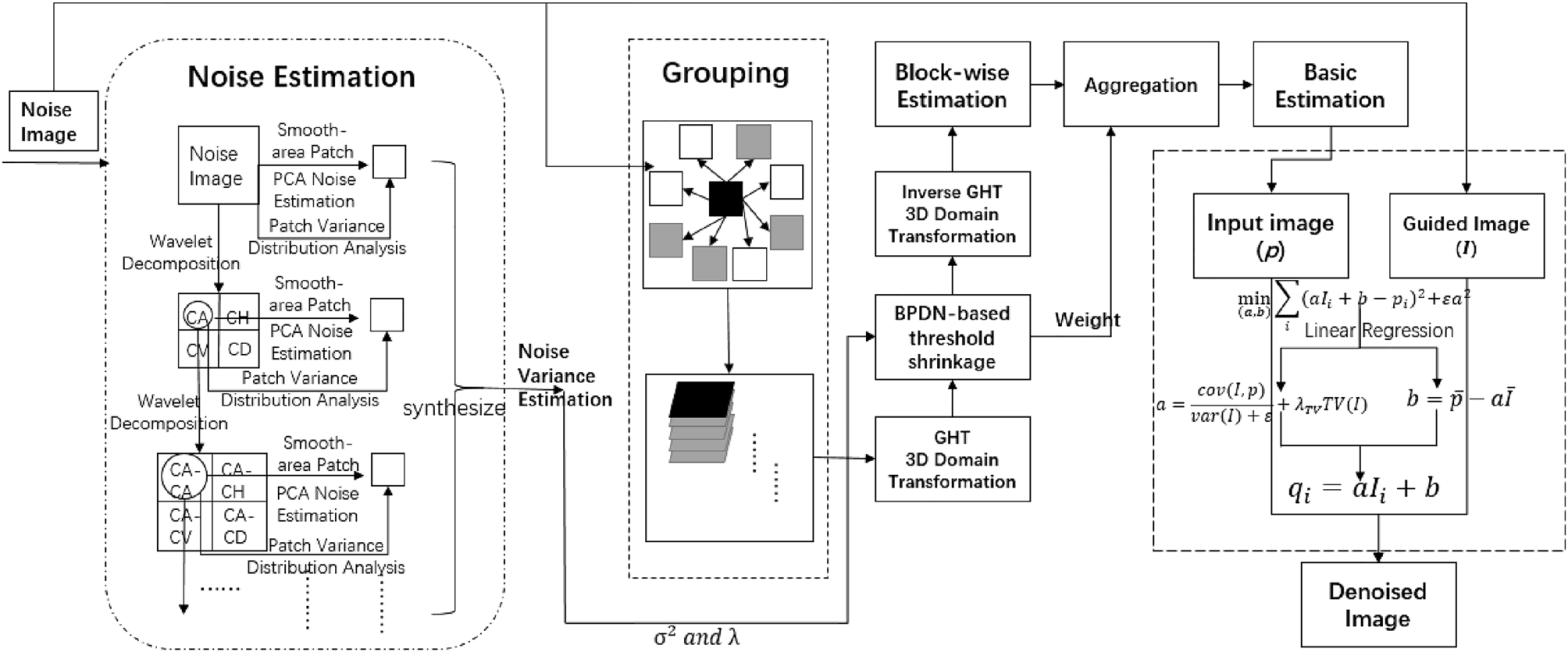
Flow chart of adaptive blind-noise image denoising method.

The proposed adaptive blind noise image denoising method consists of three parts: noise estimation, basic estimation and final denoising. To better understand the proposed method, we draw the flow chart, as shown in Fig. 6.

First, the noise variance is calculated by the improved PCA-based noise variance estimation method, which can facilitate image denoising. Then, in basic estimation, similar blocks in the image are grouped and aggregated into a 3D array, and the arrays is subjected to GHT-based domain transformation and BPDN-based threshold shrinking to filter noise coefficients. After block-matching domain transformation filtering, the processed coefficients are restored to a preliminarily denoised image by inverse transformation and aggregation. Finally, the improved guided filtering based on total variation is used to filter the remaining noise. In the filtering, the basic estimation and the noise image are used as the input image and the guided image respectively. After the second-order filtering process, the denoised image is obtained.

## Experimental results

**Figure 7:**
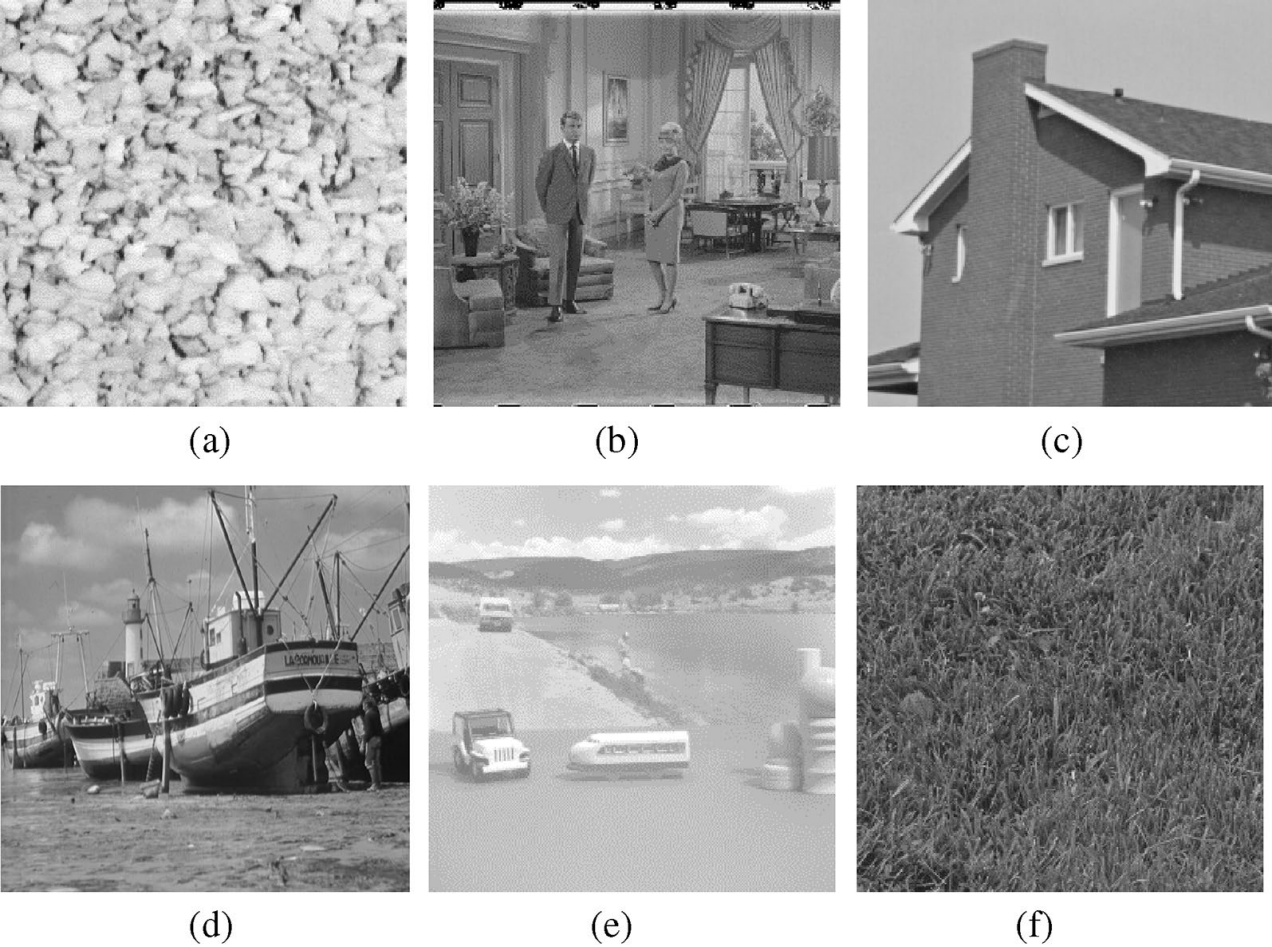
The example images. (**a**) Gravel (**b**) Couple (**c**) House (**d**) Boat (**e**) Toy Vehicle (**f**) Grass.

In order to verify the performance of the proposed blind-noise image denoising method, we use the standard images provided in The USC-SIPI Image Database to carry out the denoising experiment, some experimental test images are shown in Fig. 7. Some representative denoising methods are used to compare with the proposed method, and PSNR is used as the evaluation of denoising effect. The proposed method can be divided into two parts: noise variance estimation and image denoising. Therefore, our experiments will verify the effect of noise variance estimation and denoising effect respectively. And all the simulations are performed under the MATLAB 2018b, with Intel(R) Core(TM) i7-8750H CPU @ 2.20 GHz CPU and 16 GB RAM environment. The testing images will be corrupted by various levels of additive Gaussian, which is implemented by function ‘imnoise’.

### Results of noise variance estimation

For decades, many noise variance estimation methods have been proposed. Among them, the representative ones are Donoho robustness estimation^31^, local image block noise variance distribution analysis^32^, PCA-based noise variance estimation^33^, Laplace-based noise variance estimation^34^, and noise level estimation using weak textured patches (WTPS)^35^, which are used for comparative experiments. The comparison results are shown in Table 3, and the following conclusions are drawn from the experimental results:

1. Among the numerous estimation methods, the PCA-based noise variance estimation method and the proposed method perform well in various situations. Although the proposed method improves the accuracy of the estimation, the error still exists. The estimation method still needs further research.
2. Since the gray level of the image is limited, when the noise variance is too large, the polluted pixel value will exceed the maximum gray level, but the limitation of the gray level makes this part of the noise not manifested in the image. Therefore, the noise in the simulation experiment is sometimes not fully manifested. This part of the noise is difficult to estimate accurately.
3. If there is an image with a lot of contour and texture details, and less flat areas. When the noise value is low, the multi-layer wavelet decomposition does not distinguish the high-frequency information and noise of the image very well, and the obtained image approximates that only the first layer of information is available, so the obtained results are the same as the PCA-based estimation method.

**Table 3:** The results of various noise-variance estimation methods.

Image	Noise variance	Donoho robustness estimation	Local block noise-variance distribution	WTPS	Laplace-based	PCA-based	The proposed
House	5	12.85	6.89	6.79	8.14	5.59	5.59
	10	16.49	9.96	9.67	10.90	10.41	10.41
	15	19.17	12.16	11.96	12.98	15.08	15.08
	20	21.58	14.04	13.72	14.69	19.44	22.7
	25	23.34	15.54	15.18	16.14	23.74	27.2
	30	25.17	16.95	16.65	17.49	28.19	30.9
	35	26.55	18.09	17.65	18.48	31.59	34.7
Couple	5	10.31	7.17	7.22	7.24	3.86	4.4
	10	14.09	9.87	9.84	10.62	7.27	8.7
	15	16.73	11.67	11.74	12.66	12.33	13.3
	20	18.80	13.41	13.29	14.35	14.53	15.5
	25	20.61	14.75	14.65	15.68	19.11	27
	30	22.16	15.97	15.88	16.84	24.05	30.2
	35	23.47	17.02	17.03	17.94	22.59	29.8
Boat	5	11.89	7.52	7.56	7.98	5.4	5.3
	10	15.76	10.5	10.51	11.25	10.3	9.7
	15	18.55	12.64	12.47	13.39	15.04	15.1
	20	20.71	14.53	14.26	15.01	17.24	17.8
	25	22.75	15.73	15.52	16.53	20.9	21.3
	30	24.27	16.84	16.55	17.77	24.9	25.3
	35	25.69	18.02	17.82	18.74	30.26	30.9

The PCA-based estimation method has a good performance when the noise variance value is not high. In order to improve the efficiency of the proposed estimation method, we can directly use the PCA-based estimation method results when the noise variance value is lower than a certain threshold (the threshold is 1/0.6745 = 14.8).

### Results of block-matching domain transformation filtering

In this section, we compare the denoising results of the proposed method and BM3D, the denoising results include basic estimation and final estimation. The results of basic estimation verify the performance of the block-matching domain transformation filtering, and the results of final estimation verify the performance of the wiener filter and the improved guided filter. The smoothing factor ${\lambda }_{TV}$ of the improved guided filtering is set to − 0.1 for convenience. In experiments, we also compare the denoising results obtained using accurate noise variance values and noise variance estimates to demonstrate the impact of estimation errors. In this section, we mainly discuss the performance of block-matching domain transformation filtering. The experimental results are shown in Table 4, and the following conclusions are drawn:

1. The proposed method has better denoising performance in both basic estimation and final estimation, and the final estimation proves that the improved guided filtering can effectively preserve image information while denoising.
2. The noise is easily confused with the texture, contour and other details of the image, and it is difficult to remove noises in these areas. The improved guided filtering also inevitably damages image information while denoising.

**Table 4:** The comparison of denoising results between BM3D and the proposed.

Noise variance	Evaluation criteria: PSNR			Couple	House	Boat	Toy vehicle	Grass
$\sigma^{2} = 15$	BM3D	Basic estimation	Accuracy	21.95	22.52	21.37	22.32	20.89
			Estimation	20.51	22.01	21.43	21.34	20.93
		Final estimation	Accuracy	20.66	20.96	20.1	20.86	19.97
			Estimation	19.62	20.53	20.14	20.12	20
	The proposed	Basic estimation	Accuracy	24.75	26.52	24.18	26.32	22.26
			Estimation	23.08	25.89	24.22	24.97	22.57
		Final estimation	Accuracy	24.9	27.64	24.33	27.67	21.94
			Estimation	23	27.05	24.37	26.18	22.06
$\sigma^{2} = 25$	BM3D	Basic estimation	Accuracy	22.7	23.74	22.14	23.7	20.85
			Estimation	19.53	25.18	20.51	24.79	21.4
		Final estimation	Accuracy	21.79	22.8	21.13	22.66	20.1
			Estimation	18.36	25.08	19.15	24.41	20.91
	The proposed	Basic estimation	Accuracy	24.43	27.27	24.61	26.83	21.46
			Estimation	22.72	27.74	23.75	27.13	21.38
		Final estimation	Accuracy	24.31	27.96	24.62	27.76	21.1
			Estimation	22.98	28.24	24.04	27.93	20.97
$\sigma^{2} = 35$	BM3D	Basic estimation	Accuracy	22.82	24.53	22.19	24.3	20.14
			Estimation	18.11	23.5	21.05	22.23	20.1
		Final estimation	Accuracy	23.32	25.57	22.77	25.06	20.75
			Estimation	17.18	23.7	20.72	21.58	20.66
	The proposed	Basic estimation	Accuracy	23.6	27.05	24.1	26.21	20.63
			Estimation	21.62	26.85	23.91	25.8	20.64
		Final estimation	Accuracy	23.43	27.63	24.01	26.95	20.35
			Estimation	21.89	27.53	23.97	26.72	20.37

**Figure 8:**
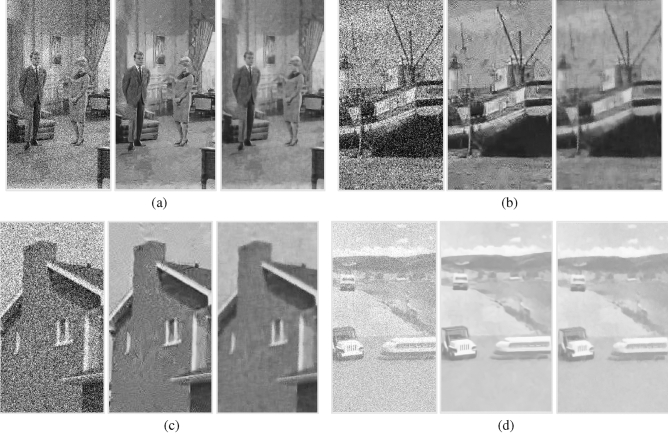
The comparison of denoised images between BM3D and the proposed (${\sigma }^{2}=35$, The left is the noise image, the middle is the BM3D denoised image, and the right is the denoised image of the proposed method) (**a**) couple (**b**) Boat (**c**) House (**d**) Toy Vehicle.

Part of the denoised image is shown in Fig. 8. It can be seen that BM3D suffers from halo artifacts. Although the proposed method reduces the halo artifacts, it also causes over-smoothing of the image. This issue deserves further research and improvement.

### Results of the improved guided filtering

**Figure 9:**
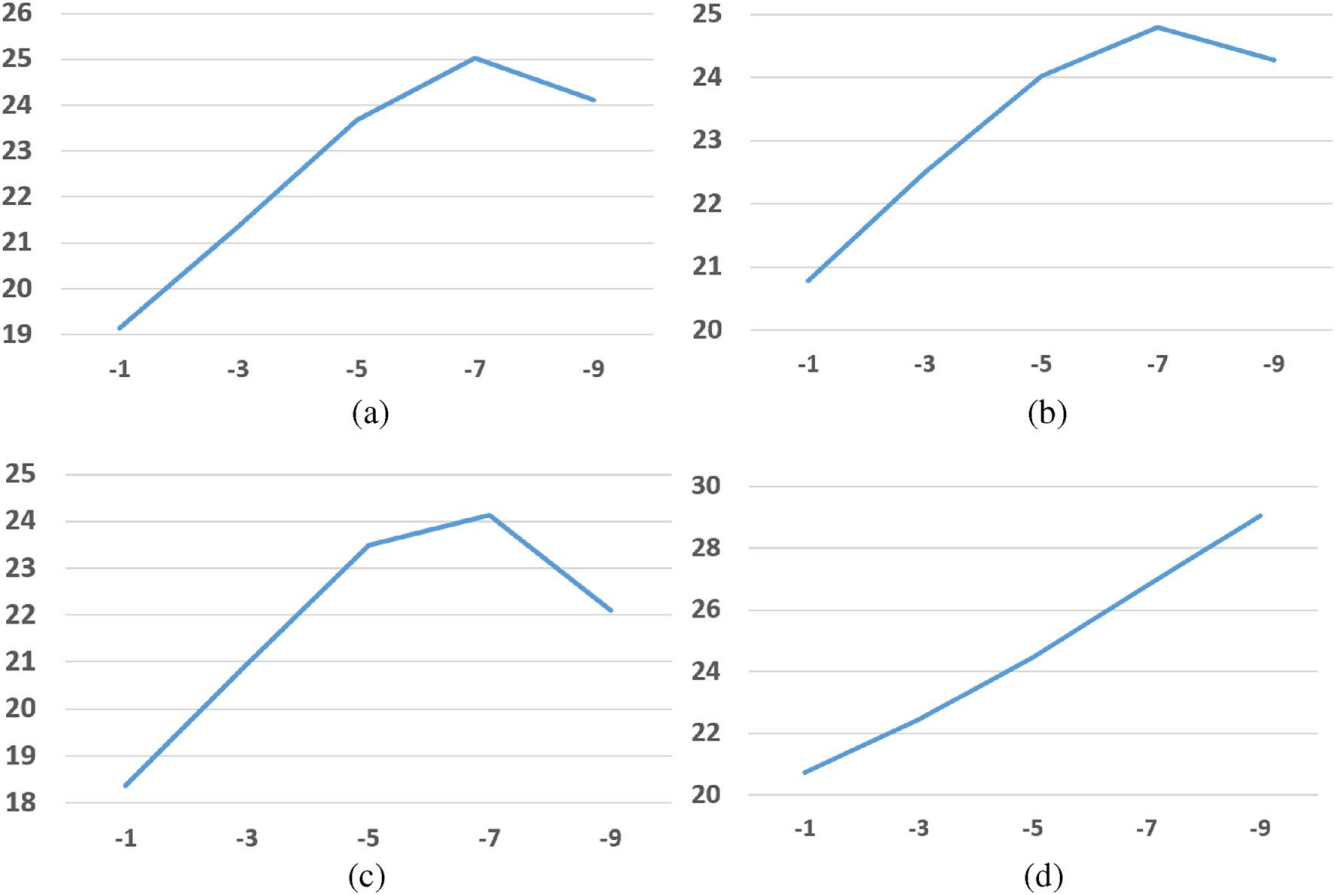
Denoising results curves of improved guided filtering with different smoothing parameters. (**a**) Couple (${\sigma }^{2}=10$) (**b**) Boat (${\sigma }^{2}=15$) (**c**) House (${\sigma }^{2}=20$) (**d**) Grass (${\sigma }^{2}=10$).

In this section, we compare the denoising effect of guided filtering and the improved guided filtering. The guided images in both guided filtering and improved guided filtering are selected to be consistent with the input images. Improved methods based on total variation require multiple iterations to obtain optimal results, and we show the effect of different smoothing factors on the results, as shown in Table 5 and Fig. 9. The bolded numbers in each row is the number with the highest PSNR. It can be concluded from the experimental results that:

1. The improved guided filtering gives a good improvement in image denoising, even with a small absolute value smoothing factor (|− 0.1|).
2. In most cases, there are smoothing factors that maximizes PSNR, and the smoothing factor is not unique, a small change in the value of smoothing factor does not change PSNR.
3. In different images, the appropriate smoothing factor is different. The use of iteration to find suitable smoothing factors makes the improved guided filtering have high computational complexity.

**Table 5:** The denoising results of the improved guided filtering with different smoothing factors.

Images		Guided filtering (PSNR)	The improved guided filtering (PSNR)					
			${\lambda }_{TV}=-0.1$	${\lambda }_{TV}=-1$	${\lambda }_{TV}=-3$	${\lambda }_{TV}=-5$	${\lambda }_{TV}=-7$	${\lambda }_{TV}=-9$
Boat	${\sigma }^{2}=5$	22.5	22.56	23.19	24.59	25.88	26.72	**26.76**
	${\sigma }^{2}=10$	19.73	19.82	20.61	22.49	24.39	**25.64**	25.38
	${\sigma }^{2}=15$	18.14	18.23	19.13	21.36	23.68	**25.02**	24.11
	${\sigma }^{2}=20$	17	17.11	18.12	20.68	23.38	**24.53**	22.7
	${\sigma }^{2}=25$	16.14	16.25	17.35	20.21	23.23	**23.91**	21.28
	${\sigma }^{2}=35$	14.9	15.03	16.29	19.63	**23.05**	22.45	18.83
Gravel	${\sigma }^{2}=5$	23.15	23.22	23.85	25.22	26.33	**26.82**	26.45
	${\sigma }^{2}=10$	20.12	20.21	21	22.87	24.6	**25.47**	24.83
	${\sigma }^{2}=15$	18.44	18.54	19.46	21.7	23.87	**24.75**	23.47
	${\sigma }^{2}=20$	17.24	17.35	18.37	20.95	23.48	**24.13**	22.09
	${\sigma }^{2}=25$	16.4	16.51	17.62	20.44	23.2	**23.46**	20.84
	${\sigma }^{2}=35$	15.11	15.24	16.5	19.8	**22.92**	22.03	18.54
Couple	${\sigma }^{2}=5$	22.84	22.89	23.41	24.66	26.06	27.6	**29.24**
	${\sigma }^{2}=10$	19.98	20.05	20.73	22.44	24.45	26.78	**29.06**
	${\sigma }^{2}=15$	18.39	18.47	19.28	21.34	23.85	26.75	**28.74**
	${\sigma }^{2}=20$	17.29	17.38	18.29	20.64	23.58	26.78	**27.52**
	${\sigma }^{2}=25$	16.4	16.51	17.5	20.12	23.46	**26.67**	25.76
	${\sigma }^{2}=35$	15.16	15.28	16.42	19.52	23.52	**25.75**	22.37
Grass	${\sigma }^{2}=5$	23.11	23.2	23.96	**25.16**	25.1	23.83	22.08
	${\sigma }^{2}=10$	20.07	20.18	21.1	22.95	**23.68**	22.68	20.74
	${\sigma }^{2}=15$	18.4	18.51	19.54	21.77	**22.9**	21.84	19.62
	${\sigma }^{2}=20$	17.19	17.31	18.44	21.01	**22.4**	21.08	18.52
	${\sigma }^{2}=25$	16.34	16.47	17.68	20.51	**22.06**	20.43	17.59
	${\sigma }^{2}=35$	15.02	15.16	16.51	19.78	**21.43**	19.08	15.84

### Results of the proposed method

**Figure 10:**
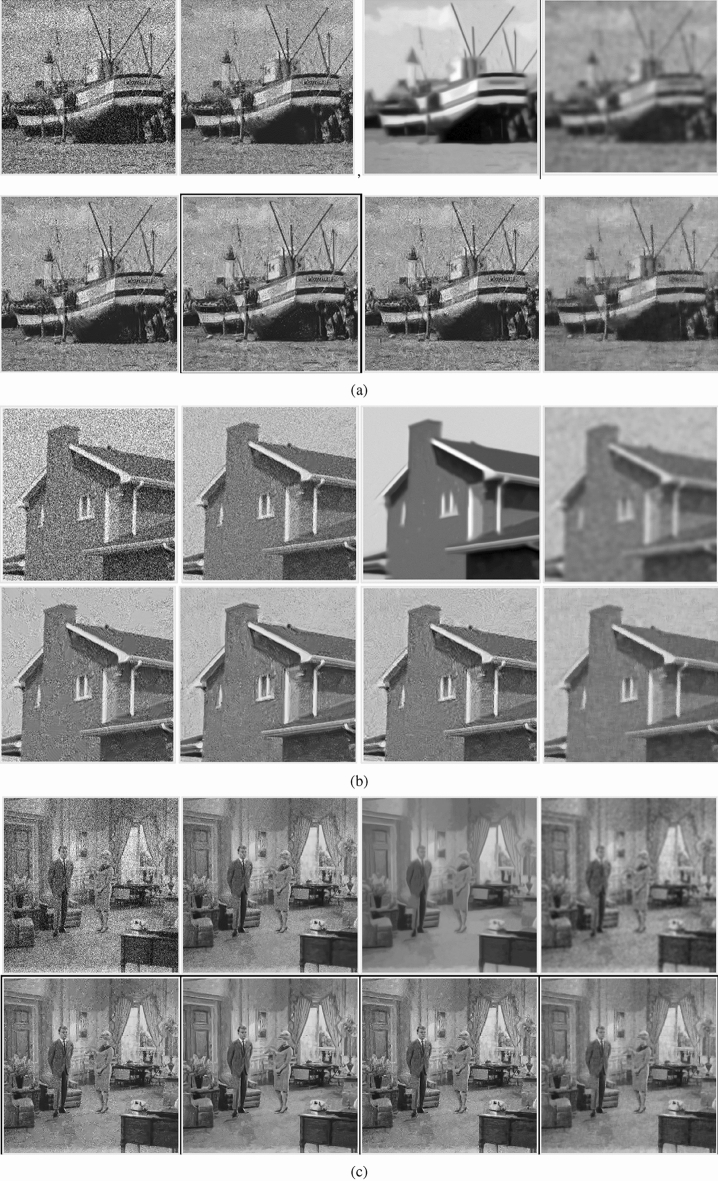
The results of various denoising methods. (**a**) Boat (σ^2^ = 25) (**b**) house (σ^2^ = 30) (**c**) Couple (σ^2^ = 35). From left to right and top to bottom are noise image, NCSR, MCWNNM, QM-based, TWSC, NLH, BM3D and the proposed method respectively.

In this section, the proposed image denoising method is compared with several representative denoising methods, such as NCSR^11^, MCWNNM^36^, TWSC^13^, Quantum mechanics-based (QM-based)^37^, NLH^38^. These methods are of great significance to the research of image denoising, and have reference value for the evaluation of denoising effect. The experimental results are shown in Table 6 and Fig. 10. The bolded numbers in each row is the number with the highest PSNR. The running times of various methods are shown in the Table 7, and the value of running time is the median value of multiple experiments.

**Table 6:** Denoising results of various methods.

Images (Noise variance $\sigma^{2}$)		Noise	NCSR	MCWNNM	QM-based	TWSC	NLH	BM3D	Proposed
Gravel	5	23.02	23.53	23.54	19.82	23.33	23.31	23.49	27.29
	10	20.05	21.22	23.34	19.77	20.85	20.58	21.19	26.37
	15	18.39	20.49	23.13	19.73	19.92	19.5	20.57	25.64
	20	17.2	20.52	22.97	19.71	19.73	19.56	20.75	25.12
	25	16.36	20.98	21.77	19.63	20.29	20.7	21.79	24.49
	30	15.69	21.63	21.73	19.6	21.39	22.86	22.87	24.35
	35	15.09	23.29	21.51	19.52	22.67	24.81	23.43	23.91
Couple	5	22.68	23.21	23.01	21.04	22.99	22.97	23.15	26.06
	10	19.88	21.13	22.82	20.95	20.72	20.71	21.08	25.58
	15	18.37	20.61	22.65	20.85	19.99	20.8	20.66	25.4
	20	17.22	20.64	22.45	20.73	19.81	21.57	20.9	24.88
	25	16.37	20.96	21.72	20.63	20.19	23.18	21.79	24.32
	30	15.67	21.46	21.56	20.54	20.9	25.45	22.76	23.81
	35	15.11	23.04	21.44	20.43	22.16	25.84	23.32	23.43
House	5	23	23.61	28.39	24.84	23.37	23.2	23.57	30.34
	10	20.03	21.41	28.1	24.74	20.96	20.47	21.37	29.32
	15	18.37	20.84	27.86	24.63	20.15	19.4	20.96	28.84
	20	17.12	20.89	27.55	24.57	19.94	19.47	21.36	28.57
	25	16.24	21.36	26.81	24.51	20.54	20.89	22.8	28.2
	30	15.53	22.14	26.56	24.36	21.72	24.83	24.49	27.76
	35	14.94	24.27	26.47	24.33	23.59	27.72	25.57	27.63
Boat	5	22.38	22.82	24.07	22.20	22.65	22.61	22.78	24.15
	10	19.67	20.73	23.83	22.13	20.38	20.13	20.7	24.75
	15	18.09	20.04	23.59	22.05	19.48	19.17	20.1	25.39
	20	16.96	20.09	23.34	22.01	19.26	19.58	20.3	24.32
	25	16.1	20.37	22.84	21.93	19.61	21.29	21.13	24.71
	30	15.47	21.02	22.66	21.82	20.51	23.63	22.31	24.39
	35	14.88	22.33	22.54	21.73	21.64	24.66	22.77	24.01
Grass	5	23.04	23.38	20.55	18.85	23.27	23.35	23.37	24.47
	10	20.02	20.87	20.37	18.83	20.64	20.5	20.87	23.08
	15	18.36	19.96	20.25	18.82	19.56	19.15	19.97	22.18
	20	17.16	19.75	20.08	18.79	19.16	18.56	19.77	21.55
	25	16.31	19.93	19.65	18.77	19.45	18.63	20.1	21.1
	30	15.58	20.2	19.56	18.74	20.08	19.67	20.43	20.65
	35	15	21.44	19.46	18.71	21.03	21.3	20.75	20.48

**Table 7:** Running time of various denoising methods (s).

Methods	NCSR	MCWNNM	QM-based	TWSC	NLH	BM3D	Proposed
Running time (s)	130.86	100.23	123.72	78.31	22.77	0.8	2.2

From the experimental results, it can be seen that in most cases, the denoising effect of the proposed method is better than other methods, and it has a lower computational complexity. For the visual quality of denoised images, both denoising and detail-preserving should be taken into account. Compared with other methods, the proposed method reduces artifacts, does not cause over-smoothing, and has a better compromise on denoising and detail preservation. However, the proposed method causes some image blurring. It is a worthy project in the further study.

## Conclusion

In this paper, a blind noise image denoising method is proposed, which consists of noise variance estimation, block matching domain transform filtering and improved guided filtering. First, we improve the PCA-based noise variance estimation method by using multilayer wavelet decomposition to obtain a more accurate noise variance estimation. Then, according to the learning and analysis of BM3D, a block-matched domain transform filter based on compressed sensing is proposed to reduce noise. Finally, we improve the denoising effect of guided filtering by optimizing the relationship model between the input image and the guided image. Experimental results show that the proposed method is competitive with many benchmark and representative image denoising methods. However, the proposed method has some shortcomings, and we will improve the performance of proposed method in future work.

## Data Availability

The datasets generated and/or analyzed during the current study are available in the [The USC-SIPI Image Database] repository, [https://sipi.usc.edu/database/database.php].
